# Detection and phylogenetic analysis of parrot bornavirus 4 identified from a Swedish Blue-winged macaw (*Primolius maracana*) with unusual nonsuppurative myositis

**DOI:** 10.1080/20008686.2018.1547097

**Published:** 2018-11-30

**Authors:** Marlene Cavaleiro Pinto, Veronica Rondahl, Mikael Berg, Erik Ågren, Júlio Carvalheira, Gertrude Thompson, Jonas Johansson Wensman

**Affiliations:** a Laboratory of Microbiology and Infectious Diseases, Department of Veterinary Clinics, Institute of Biomedical Sciences Abel Salazar, University of Porto, Porto, Portugal; b Research Center in Biodiversity and Genetic Resources, University of Porto, Vila do Conde, Portugal; c Department of Pathology and Wildlife Diseases, National Veterinary Institute, Uppsala, Sweden; d Section of Virology, Department of Biomedical Sciences and Veterinary Public Health, Swedish University of Agricultural Sciences, Uppsala, Sweden; e Department of Population Studies, Institute of Biomedical Sciences Abel Salazar, University of Porto, Porto, Portugal; f Department of Clinical Sciences, Swedish University of Agricultural Sciences, Uppsala, Sweden

**Keywords:** Avian bornaviruses, bornaviruses, Parrot bornavirus 4 (PaBV-4), proventricular dilatation disease (PDD), psittacines

## Abstract

**Background:** The genus Orthobornavirus comprises RNA viruses infecting humans, mammals, birds and reptiles, where parrot bornavirus 1 to 8 causes fatal neurological and/or gastrointestinal syndromes in psittacines. There is, to the best of our knowledge, no publication describing avian bornaviruses in pet parrots in Sweden. We aimed to identify and to produce epidemiologic knowledge about the etiologic agent associated with a history of severe weight loss and death of a Primolius maracana.**Methods and results:** The results of histopathology, immunohistochemistry and real-time RT-PCR were compatible with avian bornavirus infection. Sequencing indicated infection by parrot bornavirus 4 (PaBV-4). The genotype reported shared high identity with PaBV-4 identified from pet psittacines and from wild birds in several countries. The N gene and X protein showed genotype clusters formation. P protein revealed to be more conserved within and between species of bornaviruses. Findings suggest horizontal transmission within and between avian orders and species.**Conclusion:** There seems to be a worldwide trading without biosafety measures, hence, further disease transmission could be avoided. For screening purposes, the P gene is a good candidate as a universal target in molecular diagnostics. Wild birds may be key pieces in the puzzle of bornavirus epidemiology.

## Introduction

Bornaviruses are enveloped, 80 to 100 nm in diameter with a non-segmented genome of single-stranded negative sense RNA of around 8900 nucleotides in length [1]. In captive *Psittaciformes*, parrot bornavirus 1 to 8 cause proventricular dilatation disease (PDD) [1], a chronic and fatal disease characterized by a flaccid and dilated proventriculus impacted with feeds, and variable distension of the remaining gastrointestinal tract [2]. The infection has become relevant in the context of *Psittaciformes* housed in reserves, in breeding projects of rare species, in private collections and zoos.

In Sweden, there is extensive research published regarding mammalian bornaviruses [3–12]. However, to date, there is to the best of our knowledge, no publication that identifies and characterizes at genetic and protein levels the avian bornaviruses infecting pet parrots in Sweden.

We aimed to identify and to produce epidemiologic knowledge about the etiologic agent associated with a history of severe weight loss and death of a *Primolius maracana*.

## Material and methods

### Case history

A *Primolius maracana* female died after a history of severe weight loss and was submitted a complete standardised necropsy to the Department of Pathology and Wildlife Diseases, National Veterinary Institute, Uppsala, Sweden. The macroscopic findings reported, by an experienced pathologist, during the necropsy were strongly compatible with avian bornaviruses infection. Therefore, our approach was directed to search for the presence of avian bornaviruses. Tissue samples (adrenal gland, brain, cecum, colon, crop, heart, ileum, jejunum, kidneys, liver, lung, ovary, proventriculus, tongue and trachea) were formalin-fixed in 10% buffered formaldehyde, and a selection of tissues (brain, cecum, colon, ileum, jejunum, kidney and lung) were stored at −80°C.

### Histopathology and immunohistochemistry

The formalin-fixed samples were stained with hematoxylin and eosin (HE). Immunohistochemistry to identify T-cells (polyclonal rabbit Anti-human CD3, DAKO, A00452, dilution 1:20) and B-cells (monoclonal rabbit Anti-human CD79a, Thermo Scientific, RM-9118, dilution 1:20) was performed on parafﬁn-embedded sections, which were mounted on Vectabond (Vector Laboratories, Inc., Burlingame, CA) treated glass slides, deparaffinised in xylene and rehydrated. After inhibition of endogenous peroxidase activity with 3% hydrogen peroxide for 20 min, heat-induced epitope retrieval followed. Unspecific antigen staining was blocked with 2% bovine serum albumin for 20 min prior to incubation with the primary antibodies at room temperature for 45 min. The detection was conducted with the dextran polymer method (DAKO EnVisionTM+/HRP, DakoCytomation), the colour was developed with 3,3ʹ-Diaminobenzidine (DAB) substrate and followed by counterstained with haematoxylin.

### Real-time RT-PCR and conventional RT-PCR

From stored samples at −80ºC, total RNA was extracted as previously described [11] and Qubit® RNA BR Assay kit was used to estimate concentration (ThermoFisher Scientific/Life Technologies), according to the manufacturer’s instructions. Five-hundred micrograms were reverse transcribed using the RevertAid First Strand cDNA Synthesis kit (Thermo Scientific), with random hexamer primers, according to the manufacturer’s instructions. Real-time PCR was carried out using previously reported primers [9,11] and the Power SYBR® Green Master Mix (Applied Biosystems), according to the manufacturer’s instructions. Conventional PCR was conducted using primers previously described [13], (targeting the N (forward sense) and M (reverse sense) genes) and the Dream Taq Green Master Mix (Thermo Scientific), according to the manufacturer’s instructions. The products were visualized using a 2% agarose gel, purified with the Gene Jet Gel Extraction Kit (Thermo Scientific) and analysed by Sanger sequencing (Macrogen Europe). The sequences obtained were submitted to GenBank® database under accession numberMK192982.

### Identification and phylogenetic analysis

Phylogenetic analyses were conducted based on genes (N, X, P and M) and proteins (X and P) from bornaviruses reported in GenBank®. Strains with a nucleotide identity ≥ 98%, were included into the same cluster and the relationship between each cluster and the host species, the time of sampling and the country origin of cases was evaluated. We used BLAST® (https://blast.ncbi.nlm.nih.gov/Blast.cgi) to find regions of similarity between sequences obtained and the GenBank®, as to estimate the percent identity (id%) and E-values. Analyses were conducted in MEGA7 software [14].

## Results

### Necropsy, histopathology, immunohistochemistry, real-time and conventional RT-PCR

**Figure 1.**
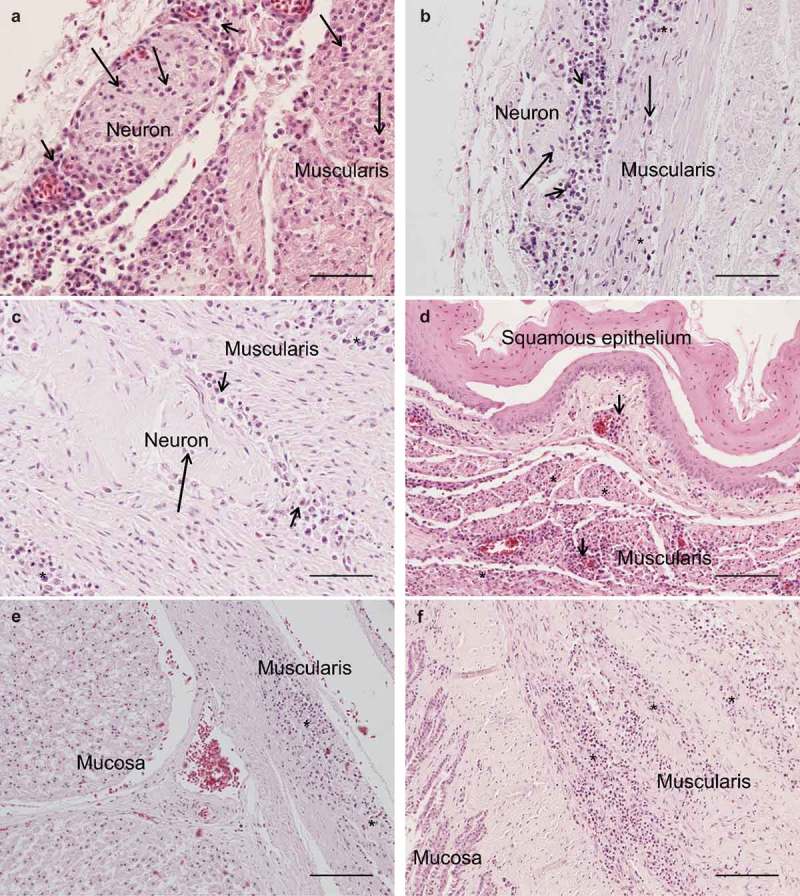
Photomicrographs of HE-stained sections. Bar = 50 µm (a, b, c) or 100 µm (d, e, f). Short arrows = perineural or perivascular lymphoplasmacytic infiltrates, long arrows = lymphoplasmacytic cells, * = diffuse intramuscular infiltrates. Lymphoplasmacytic neutritis, perineuritis and myositis in crop (a), proventriculus (b) and gizzard (c). Lymphoplasmacytic myositis in crop (d), proventriculus (e) and gizzard (f).

**Figure 2.**
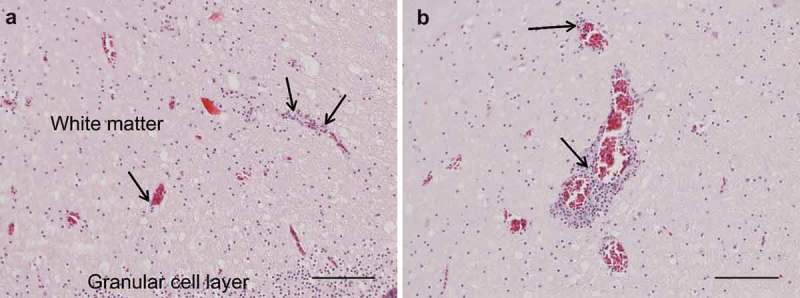
Photomicrographs of HE-stained sections, bar = 100 µm. Perivascular lymphoplasmacytic infiltrates and diffuse gliosis in the white matter of the cerebellum (A) and cerebrum (B).

**Figure 3.**
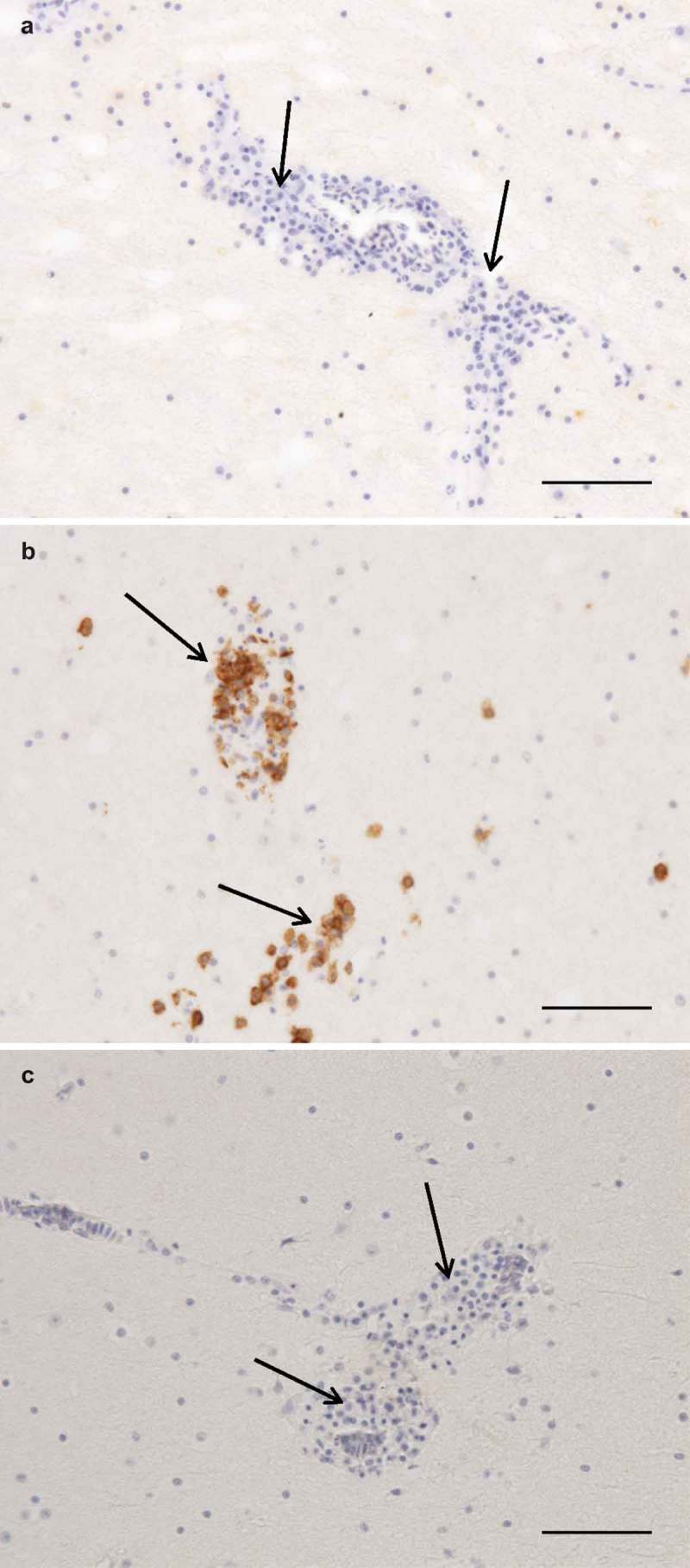
**Photomicrographs of** IHC to identify T and B cells, bar = 100 µm. Arrows = perivascular lymphoplasmacytic infiltrates. Cerebrum, negative control (a). Cerebrum, CD3-staining, numerous strongly positive (brown) cells (b). Cerebrum, CD79-staining, no positive cells (c).

The results of necropsy showed a reduced muscle mass, a severely dilated intestine and the liver and spleen were atrophic. The histopathological examination of the crop, proventriculus, gizzard and small intestine showed multifocal, mild to moderate lymphoplasmacytic intra- and perineural infiltrates (Figure 1(a,b)). There were multifocal, moderate to severe lymphoplasmacytic infiltrates in the muscularis layers of these organs (Figure 1(c), Figure 1(d), Figure 1(e,f)) associated with mild to moderate degeneration of smooth muscle cells seen as fractured cells, cell debris, pyknosis and granulated cytoplasm. In the cerebrum and cerebellum there were multifocal, mild to moderate, perivascular lymphoplasmacytic infiltrates and large areas of mild, diffuse gliosis in the white mater (Figure 2). Leucocytic infiltrates were also seen in the adrenal glands (not shown). In the other organs examined, no significant lesions were seen. Immunohistochemical detection of T- and B-cells showed that most leukocytes in the cerebrum (Figure 3) and other organs (not shown) were CD3-positive, i.e. T-cells. The samples of brain and lungs were positive by real-time PCR, and fragments of 1600 bp were obtained by conventional PCR and sequencing revealed a parrot bornavirus 4 (PaBV-4).

### Identification and phylogenetic analysis

**Figure 4.**
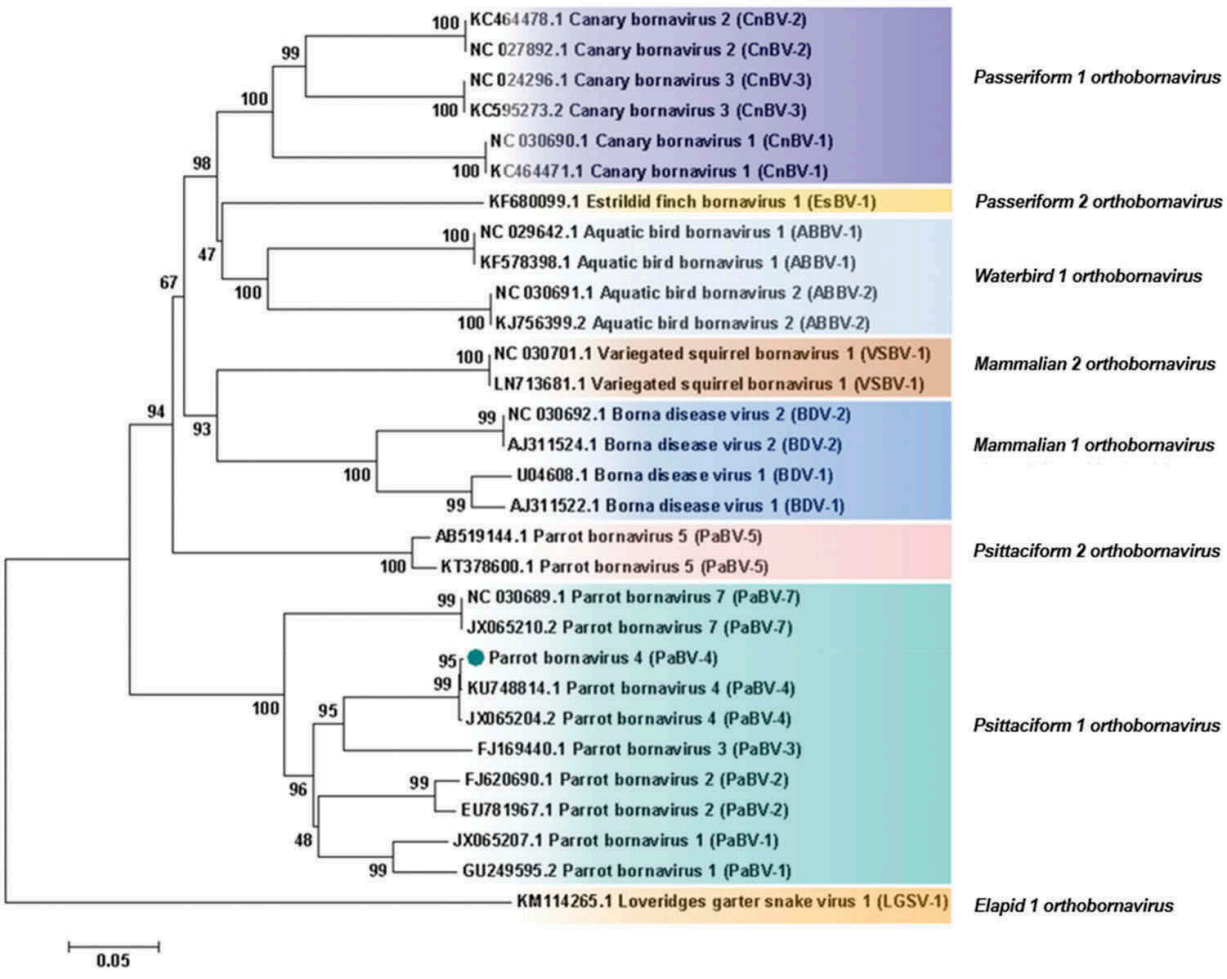
Phylogenetic relationships between the genotype identified in the present study and the selected genotypes from each *Bornavirus* species, regarding the N, X, P and M genes sequences. The evolutionary history was inferred using the Neighbor-Joining method. The percentage of replicate trees in which the associated taxa clustered together in the bootstrap test (1000 replicates) are shown next to the branches. The tree is drawn to scale, with branch lengths in the same units as those of the evolutionary distances used to infer the phylogenetic tree. The evolutionary distances were computed using the Maximum Composite Likelihood method and are in the units of the number of base substitutions per site. The analysis involved 30 nucleotide sequences. There were a total of 1527 positions in the final dataset. Evolutionary analyses were conducted in MEGA7. Sequences identified by GenBank® accession numbers, name of virus and its abbreviation name. The sequence marked with a circle was produced during this study.

**Figure 5.**
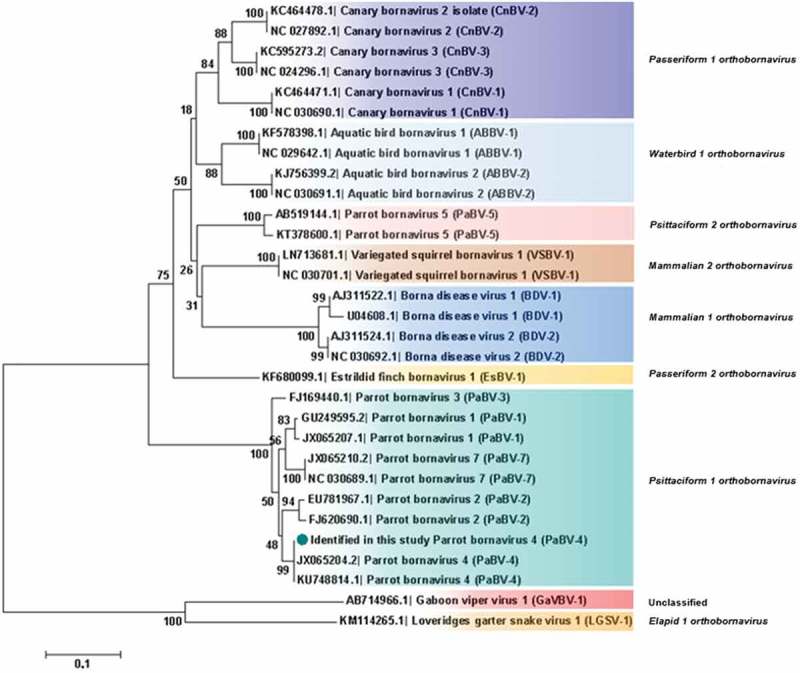
Phylogenetic relationships between the genotype identified in the present study and the selected genotypes from each *Bornavirus* species, regarding the complete P protein sequences. The evolutionary history was inferred using the Neighbor-Joining method. The percentage of replicate trees in which the associated taxa clustered together in the bootstrap test (1000 replicates) are shown next to the branches. The tree is drawn to scale, with branch lengths in the same units as those of the evolutionary distances used to infer the phylogenetic tree. The evolutionary distances were computed using the Poisson correction method and are in the units of the number of amino acid substitutions per site. The analysis involved 31 amino acid sequences. All positions containing gaps and missing data were eliminated. There were a total of 192 positions in the final dataset. Evolutionary analyses were conducted in MEGA7. Sequences identified by GenBank® accession numbers, name of virus and its abbreviation name. The sequence marked with a circle was produced during this study.

Analysis based on N, X, P and M genes revealed that PaBV-4 identified in the present study shared a higher id% with *Mammalian 2 orthobornavirus* than with some *Waterbird 1 orthobornavirus* (Figure 4) (Table 1). Analysis, based on P protein showed that PaBV-4 was more related with *Mammalian 2 orthobornavirus* than with some *Waterbird 1 orthobornavirus* and *Passeriformes 1 orthobornavirus* (Figure 5) (Table 1). The reptilian bornaviruses remain the most divergent (Figure 5) (Table 1). When analysis was based on X protein, similar phylogenetic relationship was found, however, the X protein showed lower id% than the P protein (Table 1). Therefore, phylogenetic outcomes based on protein profiles indicate the possibility of cross-reactivity between antigens of avian bornaviruses and antibodies induced by mammalian bornaviruses. A published experimental study reported that polyclonal anti-BDV-N and anti-BDV-P antisera cross-react with ABV particles [15].

**Table 1.** Identity between PaBV-4 reported and members of Genus *Orthobornavirus.*

	PaBV-4 identified in the present study						
	Genes			Proteins			
	‘N’, ‘X’, ‘P’, ‘M’			X protein		Phosphoprotein	
Bornaviruses	Id. (%) ^a^		E-value ^b^	Id. (%) ^a^	E-value	Id. (%) ^a^	E-value ^b^
**Avian ^c^**							
*Psittaciform 1 orthobornavirus*		82–99	0	75–100	2e^−63^–3e^−37^	94–100	3e^−148^–8e^−82^
*Psittaciform 2 orthobornavirus*		71–72	0	53	1e^−28^–1e^−27^	69	6e^−87^
*Passeriform 1 orthobornavirus*		70–71	0	51–57	4e^−31^–3e^−27^	68–70	5e^−96^–8e^−96^
*Passeriform 2 orthobornavirus*		71	0	63	3e^−36^	70	8e^−82^
*Waterbird 1 orthobornavirus*		69–71	0–6e^−179^	54–56	9e^−30^–4e^−27^	68–69	4e^−85^–3e^−83^
**Mammalian**							
*Mammalian 1 orthobornavirus*		69	2e^−153^–9e^−139^	45–49	5e^−23^–3e^−22^	62–64	4e^−95^–6e^−93^
*Mammalian 2 orthobornavirus*		70	5e^−161^	52	1e^−25^	69	1e^−95^
**Reptilian ^e^**							
*Elapid 1 orthobornavirus*		68	6e^−31^	37	1e^−8^	43	1e^−50^
Gabbon viper virus 1 ^f^		–	–	–	–	43	1e^−45^

^a^ The highest percent identity of all query-subject alignment given by Basic Local Alignment Search Tool (BLAST®).

^b^ The best (lowest) Expect value (E-value) of all alignments from that database sequence given by BLAST®.

^c^ Avian bornaviruses sequences considered in the analysis were the same used to construct the phylogenetic trees.

^d^ Mammalian bornaviruses sequences considered in the analysis were the same used to construct the phylogenetic trees.

^e^ Reptilian bornaviruses sequences considered in the analysis were the same used to construct the phylogenetic trees.

^f^ The database of the GenBank® only reported information for the P gene/protein.

**Figure 6.**
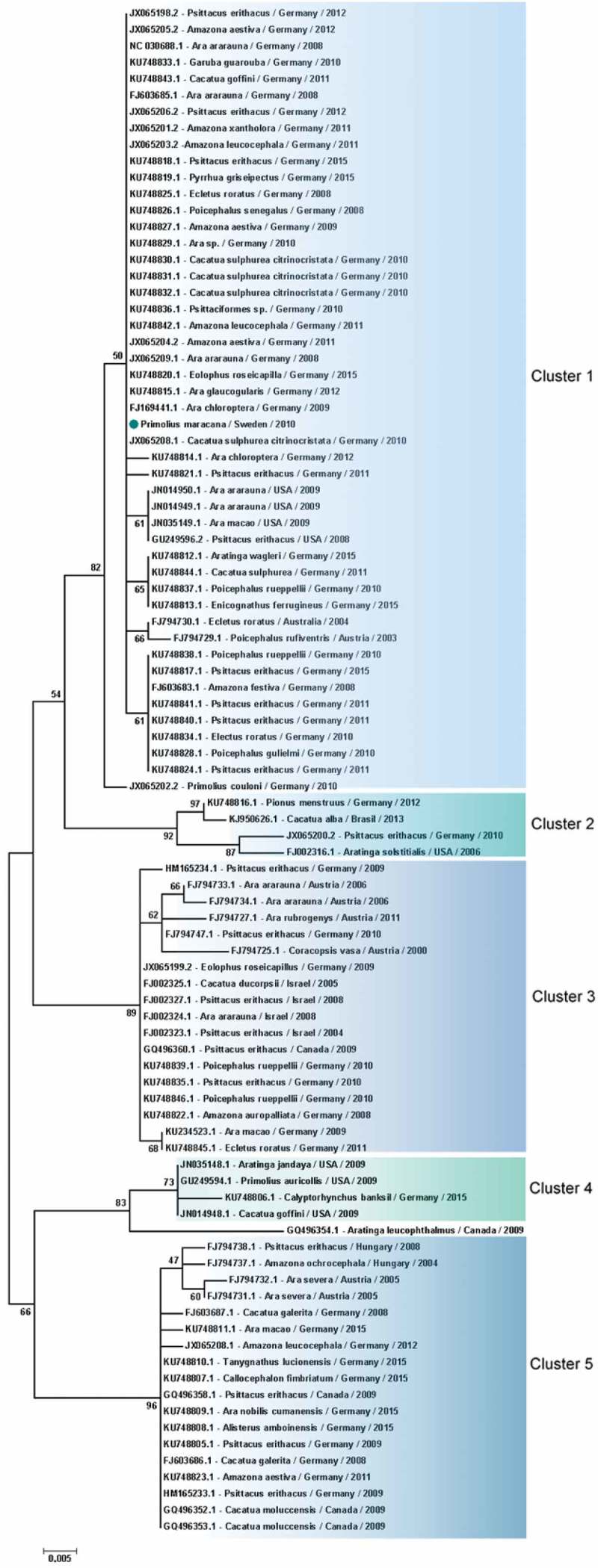
Clusters of PaBV-4 N nucleotide sequences identified from *Psittaciforms*. The evolutionary history was inferred using the Neighbor-Joining method. The percentage of replicate trees in which the associated taxa clustered together in the bootstrap test (1000 replicates) are shown next to the branches. The tree is drawn to scale, with branch lengths in the same units as those of the evolutionary distances used to infer the phylogenetic tree. The evolutionary distances were computed using the Maximum Composite Likelihood method and are in the units of the number of base substitutions per site. All positions containing gaps and missing data were eliminated. There were a total of 305 positions in the final dataset. Evolutionary analyses were conducted in MEGA7. PaBV-4 sequences are identified with GenBank® accession numbers, with the name of the host in Latin, country origin and time of sampling. The sequence marked with a circle was produced during this study.

**Figure 7.**
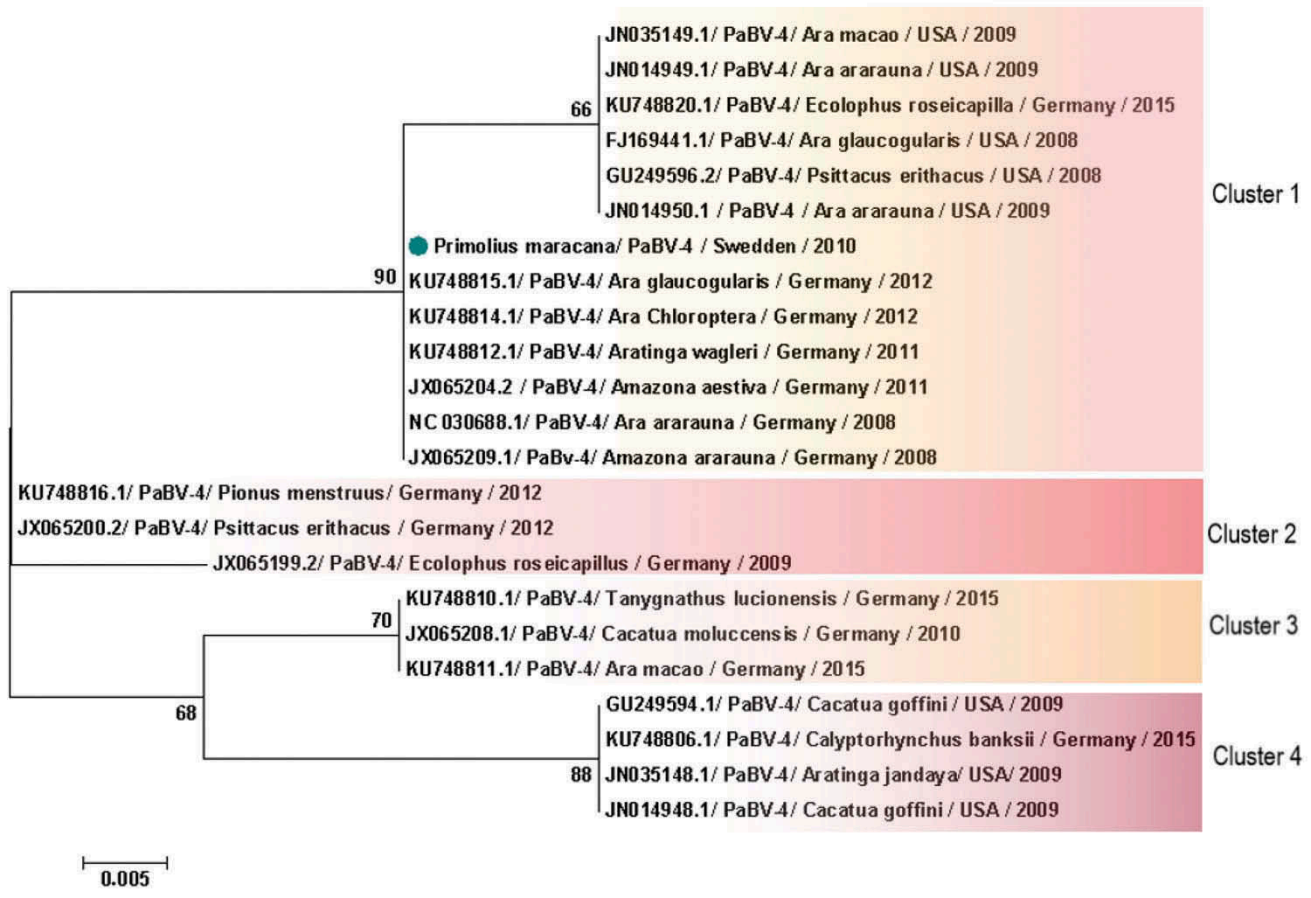
Clusters of PaBV-4 X protein sequences identified from *Psittaciforms*. The evolutionary history was inferred using the Neighbor-Joining method. The percentage of replicate trees in which the associated taxa clustered together in the bootstrap test (1000 replicates) are shown next to the branches. The tree is drawn to scale, with branch lengths in the same units as those of the evolutionary distances used to infer the phylogenetic tree. The evolutionary distances were computed using the Poisson correction method and are in the units of the number of amino acid substitutions per site. The analysis involved 23 amino acid sequences. All positions containing gaps and missing data were eliminated. There were a total of 85 positions in the final dataset. Evolutionary analyses were conducted in MEGA7. PaBV-4 sequences are identified with GenBank® accession numbers, with the name of the host in Latin, country origin and time of sampling. Sequences identified by GenBank® accession numbers, name of virus and its abbreviation name. The sequence marked with a circle was produced during this study.

When analysis was restricted to PaBV-4 N gene, 16 sequences from Japan were excluded, avoiding bias introduction, due to the small size of the sequence in the final dataset. Therefore, 93 partial PaBV-4 N gene sequences analysed were divided in 5 genotype clusters (Table 2) (Figure 6). One sequence shared < 97% of identity and was not included in the clusters (Figure 6) (Table 2). Analysis based on P protein showed absence of clusters, while based on X protein formed 4 clusters (Figure 7) (Table 2). One sequence was not included in any cluster because it shared < 97% (Figure 6); however, according to the analysis based on the N gene, it was reported to belong to cluster 3 (Figure 7). In genetic and protein approach there was a homogeneous distribution of host species, sampling time and geographic origin of cases, by clusters (Figures 6 and 7).

**Table 2.** Identity between parrot bornavirus 4 (PaBV-4) worldwide distributed.

	Gene					Proteins						
	‘N’			X protein					Phosphoprotein			
	Id. (%) ^a^	E-value	n ^c^		Id. (%) ^a^		E-value ^b^	n		Id. (%) ^a^	E-value ^b^	n ^c^
**PaBV-4 worldwide distributed**												
All sequences	95–100	4e^−51^–1e^−48^	93		93–100		4e^−51^–1e^−48^	23		99–100	4e^−51^–1e^−48^	23
**Between clusters of PaBV-4**												
Cluster 2 vs. 1	97	0–1e^−147^	–		97–98		1e^−62^–1e^−62^	–		–	–	–
Cluster 3 vs. 1	96–97	0–3e^−149^	–		95		2e^−52^	–		–	–	–
Cluster 4 vs. 1	96	0	–		93–94		4e^−51^–1e^−48^	–		–	–	–
Cluster 5 vs. 1	95–97	0–4e^−152^	–		–		–	–		–	–	–
**Within PaBV-4 clusters**												
Cluster 1	99–100	0–1e^−162^	48		99–100		2e^−63^–3e^−62^	13		–	–	–
Cluster 2	99	0	4		100		2e^−63^	2		–	–	–
Cluster 3	99–100	0–7e^−180^	18		100		2e^−63^	3		–	–	–
Cluster 4	99	0	4		100		3e^−63^–9e^−61^	4		–	–	–
Cluster 5 ^e^	98–99	0–8e^−179^	18		–		–	–		–	–	–

^a^ The highest percent identity of all query-subject alignment given by Basic Local Alignment Search Tool (BLAST®).

^b^ The best (lowest) Expect value (E-value) of all alignments from that database sequence given by BLAST®.

^c^ The sample size of each cluster.

^d^ Several parrot bornavirus 4 (PaBV-4), available from GenBank®, with complete sequences on X and P proteins were the same used to construct the phylogenetic trees.

^e^ The cluster 5 was formed only when the analysis was based on a segment of the N gene.

## Discussion

This is the first published report that identifies and phylogenetically characterizes an avian bornavirus infecting a pet parrot in Sweden. The PaBV-4 identified in the present study is more related with the zoonotic *Mammalian 2 orthobornavirus* than with some avian bornaviruses.

We have not searched for other viruses, however, during the standardised necropsy the macroscopic findings observed were strongly compatible with infection by avian bornaviruses. Lymphoplasmacytic ganglioneuritis and mild encephalitis were observed during histopathological and immunohistochemistry examinations. This is similar to the gastrointestinal lesions seen in experimentally infected cockatiels, which had lymphoplasmacytic infiltrates in the tunica muscularis of the ventricles and mild encephalitis [16]. Moreover, in a published study, cockatiels were experimentally infected with a virulent PaBV-4 strain, and the authors propose that this may have contributed to the increased immune response and inflammation seen in these animals [16]. A similar pathogenesis is therefore possible in the presented case, since the Swedish bird, additionally, developed an unusual presentation characterized by a severe lymphoplasmacytic myositis in the gastrointestinal tract. However, the experimentally infected cockatiels did not have myocyte degeneration, and much less involvement of the remaining gastrointestinal tract, as compared to the current case. In mammals, both microglial activation and CD8 + T cells contribute to the development of brain lesions [17–20], which implies that a type IV hypersensitivity reaction is occurring, as previously suggested [16]. The dominant T cell inflammation seen in all lesions in the current case supports this suggestion.

Phylogenetic analyses based on N gene and X protein, showed that PaBV-4 clusters are not linked with geographic origin, host species and time of sampling of cases; since there are a homogeneous distribution of variables by cluster. Regarding P protein, no clusters were found, also it revealed to be more conserved within all *orthobornavirus* species, than X protein. Similar findings were reported for N gene by a previous study [21] however, our approach was more comprehensive. Additionally, phylogenetic analyses suggest horizontal transmission within and between avian orders and species (Figures 6 and 7). The exact route of horizontal transmission and risk factors remain controversial [22], more studies are needed to fill this gap in knowledge, which will allow to establish preventive measures to manage the outbreaks.

Despite not meeting the criteria to be included in phylogenetic analysis, we evaluated PaBV-4 from captive psittacines of South Africa (accession no.: FJ002341, FJ002330 and GQ496351), Italy (accession no.: HM565487), Spain (accession no.: HQ728250 and HQ728251) and Japan (accession no.: AB744683 and AB744682), as well as from wild birds of Japan (accession no.: AB842432, AB842437, AB842439 to AB842441, AB842450 to AB842455, AB842464, AB842466, and AB842476 to AB842478). Therefore, the PaBV-4 identified in the present study shared a high genetic similarity with genotypes found in captive psittacines (id% = 93–100; E-value = 0–7e^−82^) and in wild birds (id% = 80–98%; E-value = 9e^−81^–5e^−40^). The findings suggest that wild birds could play a role in introduction of infection in captive flocks. A Swedish study suggested that wild birds are possible reservoirs of Borna disease virus 1 [7], with recently recognised zoonotic potential [23]. Therefore, birds could favour the emergence of novel strains, since they are reservoirs of avian and mammalian bornaviruses.

In conclusion, this study describes the first PaBV-4 in Sweden, a virus with worldwide distribution in captive psittacines, because of extensive trading without biosafety measures. Preventive measures are required, to improve the management of outbreaks, which have severe impacts for zoos, breeding projects of rare species and psittacines trade. Wild birds can be key pieces in the puzzle of bornavirus epidemiology. For screening purposes, the P gene is a good candidate as universal target in molecular diagnostics.

## References

[1] Tizard I, Shivaprasad HL, Guo J, et al The pathogenesis of proventricular dilatation disease. Anim Health Res Rev. 2016;17:110–9. 28155804 10.1017/S1466252316000189

[2] Payne SL, Delnatte P, Guo J, et al Birds and bornaviruses. Anim Health Res Rev. 2012;13:145–156. 23253163 10.1017/S1466252312000205

[3] Lundgren A-L. Feline non-suppurative meningoencephalomyelitis. A clinical and pathological study. J Comp Pathol. 1992;107:411–425. 1291589 10.1016/0021-9975(92)90015-M PMC7130315

[4] Berg A-L, Berg M A variant form of feline Borna disease. J Comp Pathol. 1998;119:323–331. 9807733 10.1016/s0021-9975(98)80054-6

[5] Berg AL, Dörries R, Berg M Borna disease virus infection in racing horses with behavioral and movement disorders. Arch Virol. 1999;144:547–559. 10226619 10.1007/s007050050524

[6] Degiorgis M-P, Berg A-L, Hård Af Segerstad C, et al Borna disease in a free-ranging lynx (Lynx Lynx). J Clin Microbiol. 2000;38:3087–3091. 10921984 10.1128/jcm.38.8.3087-3091.2000 PMC87193

[7] Berg M, Johansson M, Montell H, et al Wild birds as a possible natural reservoir of Borna disease virus. Epidemiol Infect. 2001;127:173–178. 11561971 10.1017/s0950268801005702 PMC2869725

[8] Johansson M, Berg M, Berg AL Humoral immune response against Borna disease virus (BDV) in experimentally and naturally infected cats. Vet Immunol Immunopathol. 2002;90:23–33. 12406652 10.1016/s0165-2427(02)00226-x

[9] Wensman JJ, Thorén P, Hakhverdyan M, et al Development of a real-time RT-PCR assay for improved detection of Borna disease virus. J Virol Methods. 2007;143:1–10. 17376545 10.1016/j.jviromet.2007.01.034

[10] Wensman JJ, Ilbäck C, Hjertström E, et al Expression of interferon gamma in the brain of cats with natural Borna disease virus infection. Vet Immunol Immunopathol. 2011;141:162–167. 21419498 10.1016/j.vetimm.2011.02.014

[11] Wensman JJ, Jäderlund KH, Gustavsson MH, et al Markers of Borna disease virus infection in cats with staggering disease. J Feline Med Surg. 2012;14:573–582. 22553310 10.1177/1098612X12446638 PMC11104187

[12] Lundgren AL, Zimmermann W, Bode L, et al Staggering disease in cats: isolation and characterization of the feline Borna disease virus. J Gen Virol. 2015;76:2215–2222. 10.1099/0022-1317-76-9-2215 7561758

[13] Weissenböck H, Bakonyi T, Sekulin K, et al Avian bornaviruses in psittacine birds from Europe and Australia with proventricular dilatation disease. Emerg Infect Dis. 2009;15:1453–1459. 19788814 10.3201/eid1509.090353 PMC2819881

[14] Kumar S, Stecher G, Tamura K MEGA7: molecular Evolutionary Genetics Analysis Version 7.0 for Bigger Datasets. Mol Biol Evol. 2016;33:1870–1874. 27004904 10.1093/molbev/msw054 PMC8210823

[15] Herzog S, Enderlein D, Heffels-Redmann U, et al Indirect immunofluorescence assay for intra vitam diagnosis of avian bornavirus infection in psittacine birds. J Clin Microbiol. 2010;48:2282–2284. 20392921 10.1128/JCM.00145-10 PMC2884495

[16] Payne S, Shivaprasad HL, Mirhosseini N, et al Unusual and severe lesions of proventricular dilatation disease in cockatiels (Nymphicus hollandicus) acting as healthy carriers of avian bornavirus (ABV) and subsequently infected with a virulent strain of ABV. Avian Pathol. 2011;40:15–22. 21331944 10.1080/03079457.2010.536978

[17] Stitz L, Sobbe M, Bilzer T Preventive effects of early anti-CD4 or anti-CD8 treatment on Borna disease in rats. J Virol. 1992;66:3316–3323. 1374805 10.1128/jvi.66.6.3316-3323.1992 PMC241109

[18] Lundgren AL, Lindberg R, Ludwig H, et al Immunoreactivity of the central nervous system in cats with a Borna disease-like meningoencephalomyelitis (staggering disease). Acta Neuropathol. 1995;90:184–193. 7484095 10.1007/BF00294319 PMC7086677

[19] Ovanesov MV, Vogel MW, Moran TH, et al Neonatal Borna disease virus infection in rats is associated with increased extracellular levels of glutamate and neurodegeneration in the striatum. J Neurovirol. 2007;13:185–194. 17613708 10.1080/13550280701258415

[20] Ovanesov MV, Ayhan Y, Wolbert C, et al Astrocytes play a key role in activation of microglia by persistent Borna disease virus infection. J Neuroinflammation. 2008;5:50–64. 19014432 10.1186/1742-2094-5-50 PMC2588577

[21] Rubbenstroth D, Schmidt V, Rinder M, et al Phylogenetic Analysis Supports Horizontal Transmission as a Driving Force of the Spread of Avian Bornaviruses. PloS One. 2016;11:1. 10.1371/journal.pone.0160936 PMC4990238 27537693

[22] Rubbenstroth D, Brosinski K, Rinder M, et al No contact transmission of avian bornavirus in experimentally infected cockatiels (Nymphicus hollandicus) and domestic canaries (Serinus canaria forma domestica). Vet Microbiol. 2014;172:146–156. 24933163 10.1016/j.vetmic.2014.05.011

[23] Acute encephalitis associated with Borna disease virus 1, Germany 2018 Rapid Risk Assessment. European Centre for Disease Prevention and Control (ECDC). cited 2018 326 Available from: https://ecdc.europa.eu/en/publications-data/rapid-risk-assessment-acute-encephalitis-associated-infection-borna-disease-virus.

